# A185 DUAL BIOLOGIC THERAPY IN A PATIENT WITH NIEMANN-PICK TYPE C AND CROHN DISEASE: A CASE REPORT AND LITERATURE REVIEW

**DOI:** 10.1093/jcag/gwab049.184

**Published:** 2022-02-21

**Authors:** A Hudson, P Almeida, H Q Huynh

**Affiliations:** Pediatrics, University of Alberta, Edmonton, AB, Canada

## Abstract

**Background:**

Since the availability of biologics and biosimilars, inflammatory bowel disease (IBD) therapy has been a rapidly expanding field. Dual biologic therapy has become a new area of interest. This is important given that clinical remission rates from biologic monotherapy are only 40% at one year, and that in Canadian children with IBD, up to 33% will need surgery within ten years. It also holds potential in those who are not likely to respond as well to traditional therapy, such as patients with genetic disorders. Mono-/poly-genic IBD and the role of genetics in IBD is an evolving field. Niemann-Pick disease type C (NPC), a neurodegenerative lysosomal storage disorder, is one such genetic disorder, and its’ associated predisposition to IBD is thought to be related to impaired destruction of intracellular bacteria within macrophages (impaired autophagy). The persistence of bacteria in the gut wall leads to an increased cytokine response.

**Aims:**

We present a case of a teenage patient with NPC and crohn colitis, who sustained clinical remission only after escalating to dual biologic therapy (anti-TNF (infliximab) and anti-IL12/IL23 (ustekinumab)).

**Methods:**

A literature review of dual biologic therapy in Pediatric IBD (all types of patients included) was also completed.

**Results:**

The patient presented with one month of abdominal pain, weight loss, and bloody diarrhea, 9 months after her diagnosis of NPC. Endoscopy revealed mild inflammation in the rectosigmoid junction and rectum, with pathology showing granulomatous pancolitis. Induction therapy was rectal 5-ASA. Subsequent flares resulted in the addition of azathioprine, and then infliximab. She was switched to ustekinumab after a flare 21 months later, which did not lead to adequate improvement, so infliximab was added back on as a dual biologic. With this combination therapy she maintained clinical, biochemical, and endoscopic remission, ongoing two years later. A literature review revealed eight studies (case series/reports only). One Pediatric patient case combined ustekinumab with infliximab, while the remaining combined vedolizumab (gut-specific anti- α _4_β _7_ integrin) with infliximab.

**Conclusions:**

In Pediatric patients with genetic disorders and IBD who are not responding adequately to biologic therapy, adding a second biologic medication with a different mechanism of action may be efficacious. Targeting both TNFa (which induces pro-inflammatory cytokines, among many other roles), and the proinflammatory cytokines themselves (IL12/23), may be important in genetic disorders with impaired macrophage function and increased cytokine response. This is a potential option even several years into treatment. To date, only case studies are available, with the majority combining vedolizumab with infliximab. Our case adds to the sparse literature on the utility of combining ustekinumab and infliximab.

**Funding Agencies:**

None

